# Arginase 1+ microglia reduce Aβ plaque deposition during IL-1β-dependent neuroinflammation

**DOI:** 10.1186/s12974-015-0411-8

**Published:** 2015-11-04

**Authors:** Jonathan D. Cherry, John A. Olschowka, M. Kerry O’Banion

**Affiliations:** Department of Pathology and Laboratory Medicine, University of Rochester School of Medicine and Dentistry, Rochester, NY 14642 USA; Department of Neurobiology & Anatomy, University of Rochester School of Medicine and Dentistry, Rochester, NY 14642 USA; Department of Neurology, University of Rochester School of Medicine and Dentistry, Rochester, NY 14642 USA

**Keywords:** Neuroinflammation, Microglia, M2, Alternative activation, Aβ, Alzheimer’s disease

## Abstract

**Background:**

Neuroinflammation has long been considered a driver of Alzheimer’s disease progression. However, experiments developed to explore the interaction between neuroinflammation and Alzheimer’s disease (AD) pathology showed a surprising reduction in amyloid beta (Aβ) plaque deposition. We sought to understand this unexpected outcome by examining microglia phenotypes during chronic neuroinflammation.

**Methods:**

Using an adeno-associated virus vector carrying hIL-1β cDNA, inflammation was induced in one hippocampus of 8-month-old amyloid precursor protein (APP)/PS1 mice for 4 weeks, while the other hemisphere received control injections. Bone marrow chimeras and staining analysis were used to identify the origins and types of immune cells present during sustained inflammation. Arginase 1 (Arg1) and inducible nitric oxide synthase (iNOS) immunoreactivity were used as markers of alternatively activated and classically activated cells, respectively, and changes in cellular uptake of Aβ by Arg1+ or iNOS+ microglia was demonstrated by confocal microscopy. To determine if an anti-inflammatory phenotype was present during neuroinflammation, RNA was extracted on flow-sorted microglia and rt-PCR was performed. Interleukin-4 injection was used to induce alternatively activated cells, whereas a minipump and intrahippocampal cannula was used to deliver an interleukin (IL)-4Rα antibody to block the induction of Arg1+ cells in the setting of sustained IL-1β expression.

**Results:**

We observed a robust upregulation of centrally derived Arg1+ microglia present only in the inflamed hemisphere. Furthermore, in the inflamed hemisphere, greater numbers of Arg1+ microglia contained Aβ when compared to iNOS+ microglia. RNA isolated from flow-sorted microglia from the inflamed hemisphere demonstrated elevation of mRNA species consistent with alternative activation as well as neuroprotective genes such as BDNF and IGF1. To explore if Arg1+ microglia mediated plaque reduction, we induced Arg1+ microglia with IL-4 and observed significant plaque clearance. Moreover, when we reduced Arg1+ microglia induction in the context of neuroinflammation using an anti-IL-4Rα antibody delivered via intrahippocampal cannula, we observed a clear correlation between numbers of Arg1+ microglia and plaque reduction.

**Conclusions:**

Together, these findings suggest that Arg1+ microglia are involved in Aβ plaque reduction during sustained, IL-1β-dependent neuroinflammation, opening up possible new avenues for immunomodulatory therapy of AD.

**Electronic supplementary material:**

The online version of this article (doi:10.1186/s12974-015-0411-8) contains supplementary material, which is available to authorized users.

## Background

Microglia are now recognized as critical regulators of brain immunity and homeostasis and are therefore likely to play important roles in neurological disorders such as Alzheimer’s disease (AD), which is characterized by the presence of chronic neuroinflammation and accumulation of the pathological protein amyloid beta (Aβ) [1]. The link between these two components has given rise to a concept known as the inflammatory cascade hypothesis. This hypothesis states that Aβ-induced neuroinflammation can increase Aβ production, which can further increase neuroinflammation, leading to a vicious cycle that promotes AD pathogenesis [2]. In order to test this idea, we previously developed a model to explore the production of the proinflammatory cytokine interleukin (IL)-1β in the context of the APPswe/PSEN1dE9 (amyloid precursor protein (APP)/PS1) AD mouse. After 1 month of sustained IL-1β-induced inflammation, we surprisingly observed reduced Aβ deposition [3], which we later showed to be independent of the timing or duration of IL-1β induction and were able to demonstrate in a second AD mouse model [4, 5]. In this context, we described elevated microglial activity and recruitment around Aβ plaques, suggesting that these cells might contribute to decreased pathology [3–5]. Interestingly, other groups have reported similar findings in AD mice using different inflammatory cytokines, indicating that this result may be a more fundamental effect of neuroinflammation [6, 7].

Many questions still remain as to how microglia exert this seemingly beneficial effect in the context of a strong neuroinflammatory response. Importantly, recent work reveals that microglia can adopt a diverse set of “activation” phenotypes based on different environmental cues [8] that parallel findings originally described in peripheral macrophages [9]. For example, inflammatory cytokines such as TNFα or IFNγ can polarize microglia towards a classical activation phenotype characterized by inflammatory cytokine production; whereas the anti-inflammatory cytokines IL-4, IL-13, or IL-10 shift microglia towards an alternative activated phenotype characterized by debris clearance and anti-inflammatory cytokine production [8]. These two phenotypes have been designated M1 and M2 microglia, respectively [10]. The inflammatory and anti-inflammatory properties of microglia do not appear to be strictly limited to either M1 or M2 polarized cells; instead, the M1 and M2 designations may rest on the extreme ends of a spectrum of microglial phenotypes [11]. Interestingly, recent evidence suggests a mixed M1/M2 phenotype is more realistic [12]. However, where the population as a whole lies on the inflammatory vs. anti-inflammatory spectrum can dictate outcomes in disease [13].

In AD, the microglial phenotype has been suggested to be a key mechanism involved in disease progression. Being the sentinel innate immune cell in the central nervous system (CNS), phagocytosis of foreign/disease substances falls under microglial jurisdiction. Thus, Aβ accumulation suggests that microglia fail to perform a normal function. Indeed, several studies have observed that exposure to inflammatory cytokines inhibits microglial phagocytosis of Aβ [14]. The concept that inflammatory microglia fail to properly respond in disease suggests an anti-inflammatory response could potentially be beneficial for disease outcome. It has been reported that anti-inflammatory cytokines such as IL-4 can result in elevated clearance of pathological proteins both in vivo and in vitro [15, 16]. However, a detailed in vivo study specifically showing that anti-inflammatory microglia are both sufficient and necessary for clearance has not been accomplished. Using a previously described recombinant adeno-associated virus serotype 2 expression vector containing the mature form of human IL-1β (rAAV2-IL1β) [17], we observed that inflammation results in the recruitment of cells capable of producing the anti-inflammatory cytokine IL-4 into the brain. This recruitment was accompanied by the emergence of an Arg1+ microglia phenotype that preferentially engulfed Aβ. Furthermore, we demonstrated that Arg1+ microglia have the potential to be a driver of Aβ plaque reduction.

## Materials and methods

### Animals

All experimental protocols were approved by the Institutional Animal Care and Use Committee at the University of Rochester, protocol 2006-161R. Heterozygous APPswe/PS1dE9 mice (stock no. 004462) and mice with a monomeric red fluorescent protein (mRFP) under control of the chicken beta actin promoter (stock no. 005884) were purchased from the Jackson Laboratory. Mice were bred in-house to accrue appropriate experimental numbers. APPswe/PS1dE9 and wild type C57BL/6 mice were aged 7–8 months before injections were performed. Mice harboring enhanced green fluorescent protein (eGFP) under control of the interleukin-4 promoter (4Get mice) (Jackson Laboratory stock no. 004190) on a Balb/c background were a generous gift from the laboratory of Deborah Fowell.

### Construction of recombinant adeno-associated virus serotype 2

The construction and characterization of rAAV2 have been previously described [17]. The final plasmid containing a CMV promoter, ssIL-1β construct, SV40 polyA tail, and inverted terminal repeats was used to produce recombinant adeno-associated virus serotype 2 using a baculovirus intermediary and S9 cells as previously described [18]. rAAV2-Phe-scFv was used as an irrelevant control viral vector; -Phe expresses a single-chain antibody against phenobarbital [19].

### Stereotactic injections

Animals received intracranial viral injections while under isoflurane anesthesia (1.75 % isoflurane in 30/70 % oxygen/nitrogen gas) using a Kopf stereotactic apparatus. Mice were secured using ear bars and a head holder. Ophthalmic ointment was applied to prevent drying of the eyes. Betadine was used to disinfect the scalp prior to incision with a scalpel. For intrahippocampal injections in APP/PS1, 4Get, and wild-type mice, a 0.5-mm burr hole was drilled 2.18 mm caudal and 1.5 mm lateral from the bregma. A 33-GA needle was lowered 2 mm over 2 min. A Micro-1 microsyringe pump controller (World Precision Instruments) was used to inject 5 μL of rAAV2-IL1β or rAAV2-Phe using the convection-enhanced delivery (CED) method resulting in delivery of approximately 1.5 × 10^8^ infection particles/mL into each hippocampus as previously performed [20]. For intracortical injections into APP/PS1 mice, a 0.5-mm burr hole was drilled 1.5 mm caudal and 3 mm lateral from the bregma. A 33-GA needle was lowered 1.8 mm. Two microliters of recombinant interleukin-4 was injected at a rate of 200 nL/min for 10 min to deliver a final dose of 100 ng. The contralateral hemisphere received saline. The burr hole was filled with bone wax (Ethicon, Somerville, NJ), and the incision closed with 5-0 Dermalon sutures (Covidien, Mansfield, MA). Betadine and topical lidocaine were applied to the top of the suture to prevent infection and for analgesia, respectively. Mice recovered in a heated area before being placed in their home cage. Animals were sacrificed 1 month post-viral injection and 5 days post-IL-4 injection.

### Intrahippocampal cannulation

Forty-eight hours before the cannula was inserted into animals, Micro-Osmotic pumps (Alzet Model 1004) and Brain Infusion Kits (Alzet 0008851) were prepared. One hundred microliters of Anti-IL-4Rα (BD 552288) or Control IgG2a κ isotype (BD 55487) at a concentration of 1 mg/mL was injected into the Alzet pump. The flow moderator and tubing was attached as per manufacturer’s instructions. Pumps were placed into sterile saline and pre-incubated at 37 °C for 48 h for priming. On the day of surgery, animals were anesthetized and the skull was prepared and drilled using the same protocol and coordinates as the intrahippocampal stereotactic injections described above. Mice received 5 μL rAAV2-IL1β in one hippocampus while the contralateral hemisphere received 5 μL rAAV2-Phe. Immediately after viral injection and removal of the needle, the cannula was attached to the primed Micro-Osmotic pump. Using the incision in the skull, the Micro-Osmotic pump was gently inserted under the skin and down the back. The cannula was then inserted into the brain using the same burr hole as rAAV2-IL1β. The cannula was slowly lowered to 1.5 mm then secured to the skull with dental glue (C&B Metabond, Stock no. S380). The contralateral burr hole was filled in with dental wax and the incision was closed with 5-0 Dermalon sutures (Covidien, Mansfield, MA). The Micro-Osmotic pump delivered 2.64 μg/day of anti-IL-4Rα or control IgG antibody. Mice were sacrificed 28 days later.

### Bone marrow chimera

Five-month-old C57BL/6 mice received two doses of 6 Gy total body irradiation separated by 4 h. We used a Shepherd Irradiator (J.L. Shepherd and Associates) with a 6000 Ci ^137^Cs source. Care was taken to shield the head to avoid neuroinflammation induced by brain radiation. Immediately after irradiation, mice received bone marrow isolated from tibias and femurs of mRFP donor mice. Each bone marrow recipient received 200 μL of suspension for a total of 1.2 million cells via tail vein injection. After an 8-week reconstitution period, mice were subject to both rAAV2-IL1β and rAAV2-Phe hippocampal injection as described above. Upon sacrifice, blood was collected for analysis of reconstitution efficiency of the donor marrow (range of 86.1–95.1 % CD45/mRFP positive cells). Briefly, whole blood was lysed with ACK Lysis Buffer (Invitrogen) for 5 min at room temperature. Following lysis, cells were washed with ×1 phosphate-buffered saline (PBS) containing 2 % fetal bovine serum (FBS), incubated in Fc block (BioLegend), and stained with CD45-APC-eFluor 780 (eBiosciences, 47-0451-80, 1:500) and Hoechst 33258 (Molecular Probes, H1318, 1:100). Samples were analyzed on a FACS LSRII (Becton Dickinson) in the University of Rochester Medical Center Flow Cytometry Core facility, and data was acquired using FlowJo (vX) for Mac.

### Tissue

Animals were anesthetized and perfused with saline and 4 % paraformaldehyde (PFA) as previously described [5]. Harvested brains were post-fixed in 4 % PFA for 2 h at 4 °C. The fixed tissue was transferred to 30 % sucrose until equilibrated then snap-frozen in isopentane at −80 °C until used for immunohistochemistry (IHC).

### Immunohistochemistry

Brains were sectioned at 30 μm on a sliding knife microtome with a −25 °C freezing stage. Sections were stored in cryoprotectant at −20 °C until processing. Antibody staining was visualized using immunofluorescent secondary antibodies bound to Alexa fluorophores (Invitrogen) at a dilution of 1:500. Primary antibodies used were mouse anti-6E10 (Covance, clone 6E10, 1:1000), goat anti-Arginase 1 (Santa Cruz, sc-18354, 1:500), rabbit anti-iNOS (Enzo, ADI-905-431-1, 1:500), rabbit anti-NeuN (Millipore, ABN78, 1:1000), rabbit anti-GFAP (Dako, Z0334, 1:3000), rabbit anti-Iba1 (Wako, 016–20001, 1:3000), rabbit anti-Cd11c (Thermo Scientific, PA1-46162, 1:500), rat anti-Ly-6B.2 (Serotec, clone 7/4, 1:1000), rat anti-CD3 (BD Bioscience, clone G4.18, 1:500), and rat anti-Lamp1 (DSHB, clone D1B4, 1:2000). For Congo red staining, Kit HT60 from Sigma-Aldrich was used.

### Quantitative Reverse Transcription Polymerase Chain Reaction (qRT-PCR)

qRT-PCR array: 7- to 8-month-old APP/PS1 and C57BL/6 mice were subjected to either bilateral intrahippocampal rAAV2-IL1β or rAAV2-Phe injection as described above. One month after virus injection, mice were sacrificed, the brain harvested, and hippocampi isolated. A single-cell suspension was achieved using a Neural Tissue Dissociation kit (Miltenyi Biotec, 130-092-628). Myelin was removed using Myelin Removal Beads II (Miltenyi Biotec, 130-096-773). Cells were washed with ×1 PBS containing 0.5 % FBS, then incubated in Fc block (BioLegend), and stained with CD45-APC (BD Pharmingen, 557672), CD11b-Alexa Fluor 488 (BD Pharmingen, 561018), and DAPI. CD45 low, CD11b+ microglia were sorted by running samples on a FACSAria IIu (Becton Dickinson) in the University of Rochester Flow Core. Figure 3a shows the gating strategy for isolating microglia. Sorted microglia were collected into lysis buffer. Immediately after sorting, samples were processed to purify mRNA using the RNeasy Mini Plus kit (Qiagen, 74134). Purified mRNA was then amplified by using NuGen PicoSLv2 (NuGen). Samples were run on a custom TaqMan Array Micro Fluidic Card (Life Technologies Cat# 4342253) using a QuantStudio 12K Flex Real-Time PCR system (Life Technologies, Cat# 4471087). Expression values were viewed and analyzed using ExpressionSuite Software (Life Technologies, Version 1.0.3). To properly control for samples that did not have detectable mRNA, an imputation algorithm was used as previously described [21].

Standard qRT-PCR: Virus injections and tissue collection were performed as described in the qRT-PCR array. Once the hippocampi were isolated, they were immediately snap frozen in −70 °C isopentane. RNA was isolated from frozen hippocampi using TRIzol (Invitrogen) and an Omni International TH tissue homogenizer according to the manufacture’s protocols. cDNA was prepared using 1 μg of RNA and SuperScript III (Invitrogen). qRT-PCR for IL-4 was conducted using predesigned primer/probe sets (Applied Biosystems). For the housekeeping gene 18S, Taqman probe/primer sets constructed with FAM as the fluorescent marker and Blackhole I quencher (Biosearch Technologies) were used as follows: from 5′ to 3′ 18S, forward primer (F), cct gga tac cgc agc tag gaa; reverse primer (R), act aag aac ggc cat gca cca; and probe (P), cgg cgg cgt tat tcc cat gac c. Standard curves were generated using serial diluted samples over at least 5 orders of magnitude. PCR reactions were performed using iQsupermix (Bio-Rad) and 1 μL of cDNA. PCR conditions were as follows: denaturation at 95 °C for 3 min, followed by 50 cycles of amplification by denaturing at 95 °C for 30 s, annealing at 60 °C for 30 s, and extension at 72 °C for 30 s. To determine relative differences in mRNA, reaction efficiency (E) was calculated from a standard curve and cycle threshold (Ct) values were transformed using the equation expression = (1 + E)^Ct^. For normalization, 18S ribosomal RNA was used as the housekeeping gene.

### IHC analysis

Brain sections were viewed with an Axioplan 2i light microscope (Zeiss). For plaque area, a ×10 lens was used. Multiple images were taken for a single section to obtain pictures of the cortex and hippocampus. Images were merged together in Slidebook (v6.0.4) and subjected to threshold analysis using the max entropy algorithm in NIH Image J (V1.49m, http://rsbweb.nih.gov/ij/). The percent area occupied by 6E10 of the cortex or hippocampus was calculated and analyzed. The plaque ratio was generated by dividing the amount of plaque area in the inflamed hemisphere by the amount of plaque area in the control hemisphere. This was performed with three hippocampal sections per mouse and then averaged together. Statistics were generated with Student’s *t* test. Cell counts and co-localization were analyzed by capturing images at ×20 magnification. Multiple images of the hippocampus were acquired and then montaged together using Slidebook. The images were transferred to NIH Image J and the GFP number or arginase 1 (Arg1) cell number was counted manually using the cell counter feature. To determine co-localization for Aβ internalization, Arg1 cell type, peripheral RFP infiltration, and GFP expression, images were counted manually by switching individual color channels to view areas of overlap. Confocal images were captured using an Olympus FV100 laser scanning confocal microscope (Center Valley, PA). Analysis of Aβ internalization, Arg1, and nitric oxide synthase (iNOS) cell number was performed using Student’s *t* test. Correlation between Arg1 cell number and Aβ plaque area was calculated using Pearson correlation coefficients.

## Results

### Alternatively activated microglia are induced after sustained IL-1β production

**Fig. 1:**
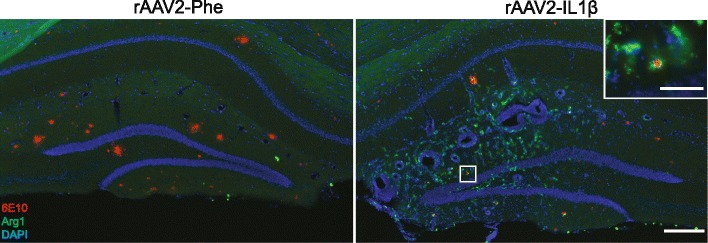
Arg1 microglia are induced after rAAV2-IL1β injection. Representative hippocampal images 4 weeks after rAAV2-IL1β injection. Images were acquired from the same histological section and show that induction of Arg1+ cells (*green*) was limited to the inflamed hemisphere (rAAV2-IL1β). *Inset* shows Arg1+ cells surrounding 6E10+ Aβ plaques (*red*). Sections were counterstained with DAPI (*blue*) to show cell nuclei. Scale bar represents 200 μm in low-power images and 50 μm in high-power inset

Using a different system of sustained IL-1β expression, we previously demonstrated increased microglia/macrophage activation using MHCII expression and morphological criteria [3, 4]. However, we did not characterize specific activation phenotypes of these microglia. Arg1 is a commonly used marker of alternatively activated microglia and macrophages [22]. We first explored if Arg1+ cells were present during sustained inflammation. rAAV2-IL1β was injected into one hippocampus of APP/PS1 mice while the contralateral hemisphere received rAAV2-Phe, which expresses a single-chain antibody to phenobarbital and was used as a control viral vector. Four weeks later, we looked for the presence of Arg1+ cells. Figure 1 shows the presence of Arg1+ cells only in the rAAV2-IL1β-injected hippocampus. Furthermore, some of these Arg1+ cells appeared to be associated with Aβ plaques (Fig. 1 inset).

**Fig. 2:**
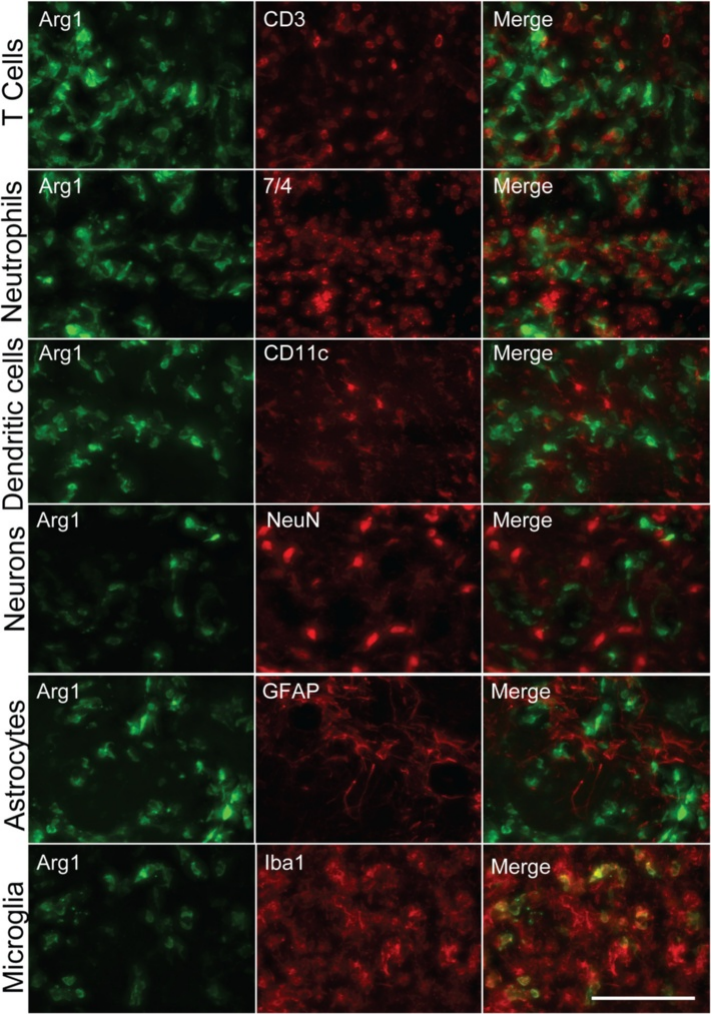
Arg1-positive cells co-localize with microglia/macrophage markers. Representative images of multiple cell types found in the brain during sustained IL-1β-induced neuroinflammation. Arg1+ cells only co-localize with Iba1. Scale bar represents 100 μm

Arg1 staining is mainly described in macrophage-like cells; however, there have been other reports suggesting infiltrating cells such as neutrophils can express it [23]. To confirm that the Arg1+ cells we observed were microglia/macrophages, dual immunohistochemistry was performed for multiple-cell markers including microglia, astrocytes, neurons, neutrophils, dendritic cells, and T cells (Fig. 2). We observed that Arg1 staining only overlapped with Iba1, indicating that microglia/macrophages were the principal cell type expressing this activation marker. Due to the differences in the origins and functions between microglia and macrophages [24], it was important to distinguish whether the Arg1+ cells arose from brain-derived microglia or infiltrating peripheral macrophages. Interestingly, we previously demonstrated that even though macrophages accumulate in our model of IL-1β expression, they are not involved in the observed clearance of Aβ, suggesting a microglia-mediated mechanism [20]. To establish the origin of Arg1+ cells, a bone marrow chimera was created. Five-month-old C57BL/6 mice received bone marrow from RFP donor mice. During the creation of chimeric mice, we avoided cranial radiation exposure by shielding the head. At 7 months of age, chimeric mice received a unilateral injection of rAAV2-IL1β and were sacrificed 1 month later. Arg1 staining was performed, and the number of Arg1+ cells that co-stained with RFP was counted (Additional file 1: Figure S1). Only 10.2 ± 3.4 % of Arg1+ cells co-labeled with RFP, suggesting that the majority (~90 %) of Arg1+ cells observed represent brain-derived microglia.

### Microglia phenotype characterization

**Fig. 3:**
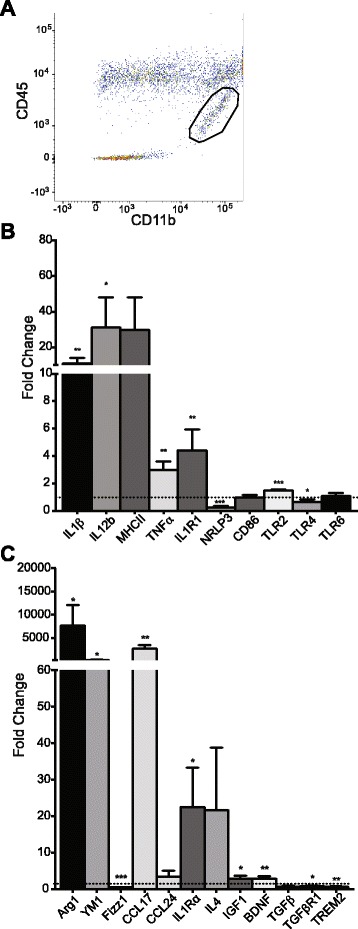
Microglial phenotype genetic profile. **a** Representative image demonstrating the gating strategy used to isolate CD11b+ CD45^low^ microglia. *Circled area* represents sorted microglia population. qRT-PCR analysis of inflammatory (**b**) and anti-inflammatory (**c**) gene expression for hippocampal CD11b+ CD45^low^ microglia during sustained inflammation. Values are normalized to the control, rAAV2-Phe-injected hemisphere, represented by the *dotted line*. Data was analyzed with Student’s *t* test. Data displayed as mean ± SEM, *n* = 4–5 animals. *p < 0.05, **p < 0.005, ***p < 0.0001

To further characterize the microglia population, multi-gene qRT-PCR was performed. First, it was necessary to separate microglia from macrophages. Therefore, inflamed hippocampi of APP/PS1 mice were dissected, CD11b+ CD45^Low^ microglia were isolated by flow cytometry (Fig. 3a), and subjected to mRNA extraction. In order to properly gauge the glial phenotype during inflammation, we chose to examine genes that could be classified as inflammatory or anti-inflammatory. For the inflammatory genes examined, we observed significant elevation in murine IL-1β, IL-12b, TNFα, and toll-like receptor (TLR)2 and a decrease in NLRP3 and TLR4 (Fig. 3b). For those genes classified as anti-inflammatory, we observed an increase in Arg1, YM1, CCL17, IL-1Ra, IGF1, and BDNF, while Fizz1, TGFβR1, and TREM2 showed decreased expression (Fig. 3c). Overall, it appears that relative to control-injected APP/PS1 mice, IL-1β expression leads to an increase in markers consistent with a mixed microglial phenotype.

### Arg1+ microglia contain Aβ in vivo

**Fig. 4:**
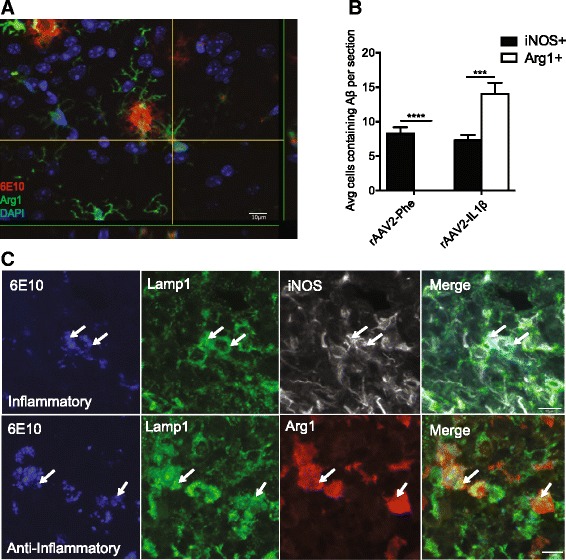
Aβ is taken up by Arg1+ microglia. **a** Representative confocal images of Arg1+ cell (*green*) containing Aβ (*red*) after rAAV2-IL1β injection. DAPI (*blue*) is used as to counter stain cell nuclei. Scale bars represent 10 μm. **b** Quantification of iNOS+ or Arg1+ microglia in the hippocampus that contain Aβ after 4 weeks rAAV2-Phe or rAAV2-IL1β injection. Data displayed as mean ± SEM, *n* = 5 animals, ***p < 0.0001, ****p < 0.00001. **c** Representative images of Arg1+ or iNOS+ microglia containing 6E10 inside the lysosome (*Lamp1*). Scale bars represent 10 μm

To determine whether Arg1+ cells associated with amyloid plaques might be involved in the clearance of Aβ, we examined tissues for co-localization of Arg1 and Aβ. Indeed, we observed multiple examples of Arg1+ cells containing Aβ (Fig. 4a). To establish whether this association was selective for Arg1+ cells, we used iNOS as a marker of inflammatory microglia [25] and counted the number of each cell type that contained Aβ (via 6E10 labeling) in hippocampal sections (Fig. 4b). In the control-injected hemisphere, we observed only iNOS+ cells containing Aβ. However, in the inflamed hippocampus, we observed a nearly twofold increase in the number of Arg1+ microglia that contained Aβ compared to iNOS+ microglia. To confirm that these microglia were engulfing and shuttling Aβ to the lysosome, we stained tissue sections with Lamp1 to examine lysosome co-location. We found that both iNOS+ and Arg1+ microglia contained Aβ in lysosomal compartments (Fig. 4c).

### IL-4-producing cells are present during inflammation

**Fig. 5:**
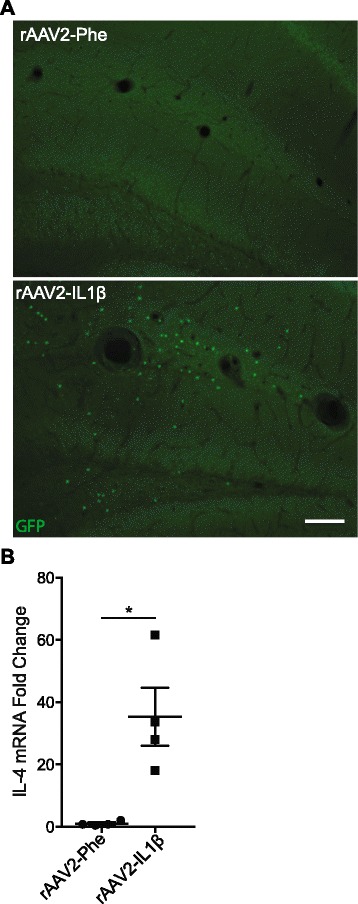
IL-4-producing cells are present in the CNS with sustained IL-1β inflammation. **a** Representative images of GFP+ cells in the hippocampus of IL-4 GFP reporter mice, 4 weeks after rAAV2-IL1β or rAAV2-Phe injection. Scale bar represents 100 μm. **b** qRT-PCR analysis of IL-4 mRNA levels for hippocampal lysates. Data was analyzed using paired Student’s *t* test. *n* = 4 animals, mean ± SEM shown, *p < 0.05

Alternative microglia induction is thought to be dependent on anti-inflammatory cytokines such as IL-4 or IL-13 [26, 27]. Therefore, we utilized an IL-4 GFP reporter mouse (4Get) to determine whether IL-4-producing cells were present in the brain after sustained IL-1β inflammation. 4Get mice were injected unilaterally with rAAV2-IL1β and sacrificed 1 month later. As shown in Fig. 5a, GFP+ cells were readily apparent in the inflamed hemisphere, while the control hemisphere was devoid of such cells. We next determined the identity of these cells using dual immunofluorescence staining with cell-specific antibodies. These results suggested that multiple cell types were capable of expressing IL-4 in our model. Of the cells that labeled with GFP, 28.7 ± 4.2 % were CD3+ T cells, 17.9 ± 5.6 % were 7/4+ neutrophils, 12.1 ± 1.9 % were GFAP+ astrocytes, and 12.1 ± 2.6 % were Iba1+ microglia (average ± SEM). To confirm the results seen in the 4Get mice and extend them to the APP/PS1 model, qRT-PCT was performed on isolated hippocampi from 9-month-old APP/PS1 mice injected with rAAV2-Phe or rAAV2-IL1β. As shown in Fig. 5b, a significant increase in IL-4 mRNA is observed 1 month after rAAV2-IL1β injection. This suggests that cells capable of producing IL-4 are present in our AD mouse model during sustained inflammation.

### Arg1+ microglia can clear Aβ

**Fig. 6:**
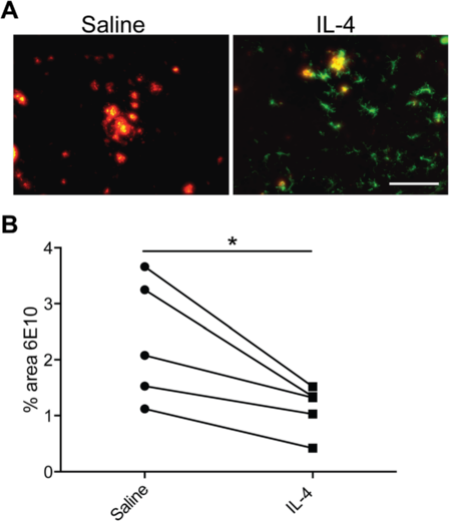
IL-4 injection leads to Arg1+ cell induction and Aβ plaque reduction. **a** Representative images of Arg1+ cells (*green*) surrounding 6E10 Aβ plaques (*red*) 5 days after intrahippocampal IL-4 injection (100 ng). Scale bar represents 100 μm. **b** Quantitative dot plot analysis of the percent area occupied by 6E10 in the saline vs. IL-4-injected sides of five mice. Data was analyzed using paired Student’s *t* test, *p < 0.05

rAAV2-IL1β-induced inflammation has diverse effects on many cell types in the CNS [17]. In order to prove that Arg1+ microglia were a critical cell type involved in plaque reduction, we sought to specifically induce them independent from chronic inflammation. Based on our observation of IL-4-producing cells (Fig. 5) and their common use to specifically induce an alternate macrophage phenotype [28], we injected 100 ng of IL-4 into the cortex of APP/PS1 mice and an equal volume of saline into the contralateral hemisphere. Five days later, we sacrificed the mice and stained tissues for Aβ using 6E10. Figure 6a depicts clear induction of Arg1+ cells in the IL-4-treated cortex. Quantification of Aβ plaque load demonstrated a reduction in the IL-4-treated cortex compared to the saline-injected hemisphere (Fig. 6b), suggesting that IL-4-induced Arg1+ microglia have the capacity to clear amyloid plaques.

### Arg1+ microglia are involved in plaque reduction

**Fig. 7:**
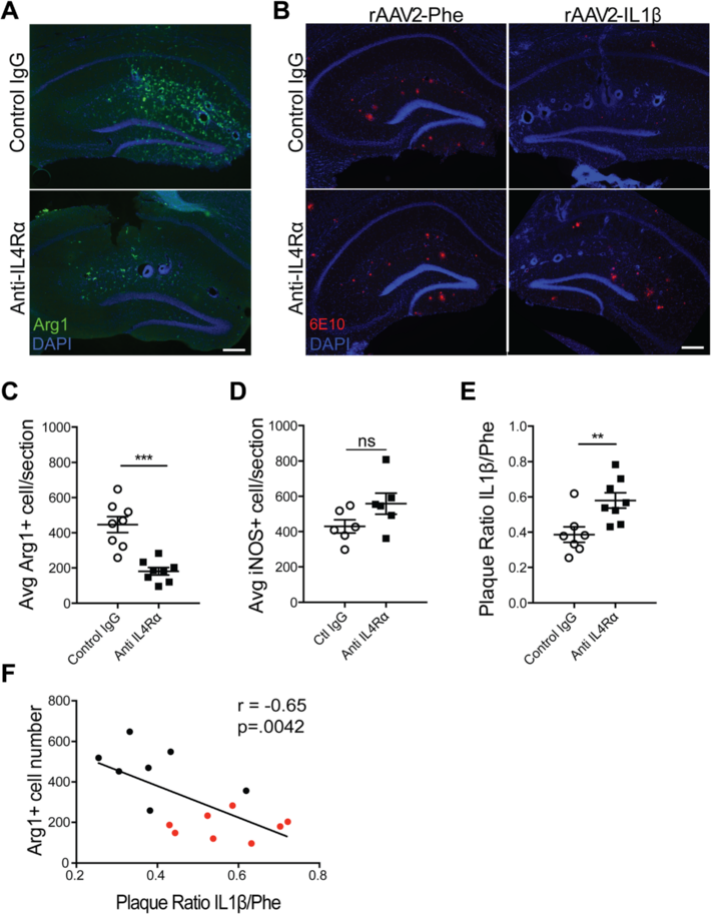
IL-4 signal blockade inhibits Arg1+ microglia induction and partially impairs Aβ plaque reduction. Representative images depict Arg1+ microglia (**a**, *green*) and 6E10 (**b**, *red*) 28 days post-injection and cannula implantation. Scale bar represents 100 μm. Quantification of Arg1+ cell number (**c**), iNOS+ cell number (**d**), and 6E10 Aβ plaque area (**e**), in the hippocampus. Data was analyzed with Student’s *t* test. *n* = 7–8 animals; mean ± SEM shown; **p < 0.005, ***p < 0.0001. **f** Correlation was determined by plotting the Arg1 cell count by plaque ratio for each animal. The plaque ratio was generated by dividing the amount of plaque area in the inflamed hemisphere by the amount of plaque area in the control hemisphere. This was performed on three hippocampal sections per mouse then averaged together. The higher the number, the more plaque is present in the inflamed hippocampus. Each *dot* represents one animal. *Black dots* denote the control IgG while *red dots* denote mice that received anti-IL-4Rα. *r* Pearson’s coefficient

Previous studies in macrophages demonstrated that IL-4-signaling inhibition impairs Arg1+ polarization [26]. Our results suggest that Arg1+ mice are necessary for IL-1β-induced plaque reduction. Therefore, to directly test this hypothesis, an osmotic minipump system was used to chronically deliver an anti-IL-4Rα antibody in APP/PS1 mice with sustained IL-1β expression. Minipumps were loaded with 100 μL anti-IL-4Rα and connected to an intrahippocampal cannula. Immediately after rAAV2-IL1β injection, cannulas were implanted 0.5 mm above the injection site in APP/PS1 mice. The contralateral hemisphere received rAAV2-Phe. A control set of APP/PS1 mice received the same rAAV2-IL1β injections, but the minipump contained an equivalent amount of control IgG. After 1 month, mice were sacrificed and brain tissues analyzed for the presence of Arg1+ cells and amyloid plaque load. We observed that IL-4Rα antibody treatment successfully reduced the numbers of Arg1+ cells in the inflamed hemisphere (Fig. 7a, c). Moreover, we observed that anti-IL-4Rα treatment attenuated the reduction of Aβ plaque load seen in rAAV2-IL1β-injected mice (Fig. 7b, e). To verify that only the anti-inflammatory response was affected, we counted the number of inflammatory microglia and found that anti-IL-4Rα treatment did not significantly alter the numbers of iNOS+ cells (Fig. 7d). Finally, we observed a significant negative correlation between numbers of Arg1+ cells and Aβ plaques across the inflamed hippocampi of all mice used in this experiment (Fig. 7f). Together, these results suggest that Arg1+ microglia are involved in plaque reduction during rAAV2-IL1β-induced inflammation.

## Discussion

Here we report that Arg1+ microglia appear to participate in Aβ plaque clearance during sustained IL-1β inflammation. These results provide a mechanism to explain our previously published findings that sustained neuroinflammation leads to plaque reduction [3, 4]. The observation that an inflammatory stimulus results in an anti-inflammatory or beneficial phenotype seems contradictory at first. However, the concept of negative-feedback mechanisms is well-established in inflammatory states, which are kept in check via upregulation of anti-inflammatory factors via multiple mechanisms [29]. Furthermore, the idea that IL-1β does not directly reduce Aβ is supported in a recent publication by Heneka et al., where APP/PS1 mice were crossed to NLRP3(−/−) mice that lack IL-1β production [30]. This mouse demonstrated elevated alternatively activated microglia markers, greater microglial Aβ phagocytosis, and reduced plaque load compared to control APP/PS1 mice. These findings are very similar to ours, suggesting that direct actions of IL-1β do not explain the reduction in Aβ observed in our model. It is also unlikely that IL-1β directly drives alternative activation of microglia to express Arg1 [31]. Our observations are consistent with a model by which sustained neuroinflammation generated by rAAV2-IL1β recruits cells capable of producing Th2 cytokines to the CNS in order to quell the inflammation and mitigate possible damage. In addition, our experiments suggest that endogenous brain cells may also locally produce IL-4 in the setting of sustained neuroinflammation. One caveat to our approach is the use of an IL-4 reporter mouse or qRT-PCR analysis, which does not provide direct evidence of IL-4 protein production. However, a recent publication from the laboratory of Jonathan Kipnis uses a different IL-4 reporter system that can identify bona fide IL-4 protein producer cells [32]. They observed the infiltration of IL-4-producing T cells after two different CNS injuries. Furthermore, their findings implicate these IL-4-producing T cells in neuroprotection. While the end points are different, their results and ours both underscore an important protective mechanism for homeostasis in CNS disease.

The induction and beneficial roles of alternatively activated microglia have been topics of debate for several years. In the context of neuroinflammation, we observed twice as many Arg1+ microglia containing Aβ than iNOS+ microglia (Fig. 3). Although we have not directly conducted Aβ phagocytosis assays with Arg1+ and iNOS+ cells, our results are consistent with several other studies indicating that inflammatory microglia are poor phagocytes of Aβ [14, 16, 33]. There is some evidence that inflammatory microglia can take up Aβ, but cannot properly clear it [34]. This concept has been coined “frustrated phagocytosis” and has been implicated as a mechanism for plaque accumulation in AD [35]. This idea can possibly explain the results observed in Fig. 4b. Even though we observed iNOS+ cells containing Aβ in the control non-inflamed hemisphere, Aβ plaque reduction was only seen when Arg1+ cells were present. This is consistent with in vitro findings that IL-4-treated microglia are potentially more efficient phagocytes [33], as well as an in vivo finding in which elevated Arg1 and reduced iNOS mRNA correlated with greater Aβ microglial phagocytosis and reduced amyloid plaque load [30]. The exact mechanism behind this is still unknown; however, alternatively activated cells induced by IL-4 have a lower lysosomal pH, which results in greater proteolytic capacity [36].

Support for the original idea of polarized microglial phenotypes largely came from in vitro experiments in peripheral macrophages using single cytokines and examining expression of a limited set of genes. Recent work has clearly demonstrated that microglia have unique genetic differences from macrophages and that the in vivo environment is more complex than revealed by tissue culture [24, 37]. In efforts to better characterize the microglial population in our experimental conditions, we ran a multi-gene qRT-PCR array (Fig. 3). Originally, we hoped to use the M1/M2abc classification system [38, 39] to better understand potential functions of these microglia. However, we observed a mixed phenotype of cells that expressed both inflammatory and anti-inflammatory markers, a finding consistent with other observations in AD mouse models [39, 40]. For example, proposed M2 markers such as Arg1 and YM1 were elevated, but we also observed increases in IL-1β and TNFα. Furthermore, we found that other M2 markers such as Fizz1 and TREM2 were decreased. The decrease in TREM2 was particularly surprising given recent reports that TREM2 loss of function mutations are a risk factor for AD [41]. However, some have argued that loss of TREM2 results in amelioration of Aβ pathology [42]. Additional studies would be required to determine whether changes in TREM2 expression are related to the reduction of plaque that we see in our model.

The mixed phenotype observed by mRNA profiling could be explained due to sampling several different populations of microglia within brain tissue. Indeed, immunofluorescent staining results shown in Fig. 4 suggest the presence of both inflammatory and anti-inflammatory microglia. However, it is possible that individual cells still express a mixed phenotype. To explore this fully, we would need to examine microglial cell phenotype at the single-cell level. Interestingly, work from the laboratory of Christine Hsieh suggests that even if unique populations of Arg1+ macrophages are sorted after traumatic brain injury, a mixed phenotype is still observed [43]. This suggests the possibility that cells with mixed phenotypic markers were present in our model. Thus, we have elected to define this cell population by Arg1+ expression rather than defining it as M1 or M2.

Due to the complexity and likely flux of microglial phenotypes, it is difficult to prove that one microglia subtype is involved in Aβ plaque clearance. However, the work described here presents a possible mechanism whereby Arg1+ microglia might participate in in vivo Aβ clearance. This data is in agreement with previously published reports using different AD mouse models, which demonstrated an association between IL-4-induced Arg1+ microglia (via either AAV-IL-4 or acute IL-4 injection) and plaque clearance [15, 44]. This does not rule out other mechanisms of Aβ clearance, as we did not see a complete inhibition during antibody treatment. Furthermore, it will be important to consider the effect of IL-4 on other cell types in the CNS. In particular, many other cell types, including those we observe in the inflamed state (Fig. 2), possess the IL-4Rα [45–49] and are therefore potential targets of receptor blockade. Future experiments will be necessary to determine the roles of other cell types in promoting Aβ clearance. Nevertheless, from a therapeutic perspective, it is promising that we associated amyloid clearance with a microglial population marked by Arg1 expression. Interestingly, the data from these studies and several others like it have spurred clinical studies [50, 51] aimed to switch inflammatory microglia to a more beneficial phenotype. PPARγ agonists [52], bexarotene [53], and glatiramer acetate [54] are just a few of the therapeutics currently being tested to induce beneficial microglial phenotypes during disease; many of these approaches impact at least some aspect of the IL-4-signaling cascade [8]. Our observations provide compelling evidence that IL-4-dependent Arg1+ microglia are involved in Aβ plaque reduction. This study helps to define one mechanism of microglia-dependent Aβ clearance; in particular, our approach utilizing an IL-4Rα antibody leaves the inflammatory response intact, demonstrating that anti-inflammatory microglia are necessary for Aβ clearance. Being able to tease glial phenotypes apart during in vivo neuroinflammation is an important step in understanding functions of unique cell phenotypes that might be elicited by other approaches.

However, not all see beneficial outcomes with anti-inflammatory cytokine treatment. Chakrabarty et al. reported an opposite effect of IL-4 on AD [55], with sustained AAV-IL-4 expression leading to increased plaque accumulation. It is not clear why there are conflicting outcomes, but different transgenic mice might underlie the contradictory results as well as different approaches, durations, and doses of IL-4 used. Furthermore, recent reports indicate that IL-10 is detrimental in AD, with IL-10^−/−^ mice crossed to APP/PS1 mice showing reduced Aβ plaque pathology [56]. This mechanism was attributed to an elevation in phagocytic ability of microglia. Clearly, more work needs to be performed to better understand the relationships between inflammatory and anti-inflammatory responses, the plasticity of microglia, and their roles in disease.

In conclusion, we observed Arg1+ microglia during sustained IL-1β neuroinflammation. These cells appear to arise from the recruitment of cells capable of producing Th2 cytokines, such as IL-4, that alternatively activate a population of endogenous microglial cells, presumably to maintain homeostasis. Interestingly, these Arg1+ microglia appeared to be more adept at Aβ phagocytosis compared to inflammatory iNOS+ microglia, suggesting that they may underlie our observations of plaque clearance when IL-1β is overexpressed. Indeed, our data showing that IL-4 alone was sufficient to mediate Arg1+ cell induction and reduced plaque load, combined with evidence that blocking IL-4 signaling partially abrogated the effects of IL-1β on Arg1+ cell induction and plaque clearance, supports a model whereby Arg1+ cells participate in plaque clearance in the setting of sustained neuroinflammation. Future studies with in vivo imaging of the interaction between these cells and Aβ deposits could be performed to provide further evidence for this mechanism.

## Additional file

Additional file 1: Figure S1. Bone marrow chimeric mice demonstrate minimal overlap between peripheral donor RFP+ cells and Arg1+ cells. Representative image depicting RFP+ donor cells (*red*) and Arg1+ cells (*green*) 1 month after AAV-IL1β injection. Scale bar represents 50 μm.

## Abbreviations

AAV: adeno-associated virus
AD: Alzheimer’s disease
APP: amyloid precursor protein
Arg1: arginase 1
Aβ: amyloid beta
CED: convection-enhanced delivery
CNS: central nervous system
eGFP: enhanced green fluorescent protein
IL: interleukin
iNOS: nitric oxide synthase
mRFP: monomeric red fluorescent protein
TLR: toll-like receptor
