# Supplementary figures and images for: Arginase 1+ microglia reduce Aβ plaque deposition during IL-1β-dependent neuroinflammation
Source: J Neuroinflammation. 2015 Nov 4;12:203. doi: 10.1186/s12974-015-0411-8 (PMC4634600; doi:10.1186/s12974-015-0411-8)

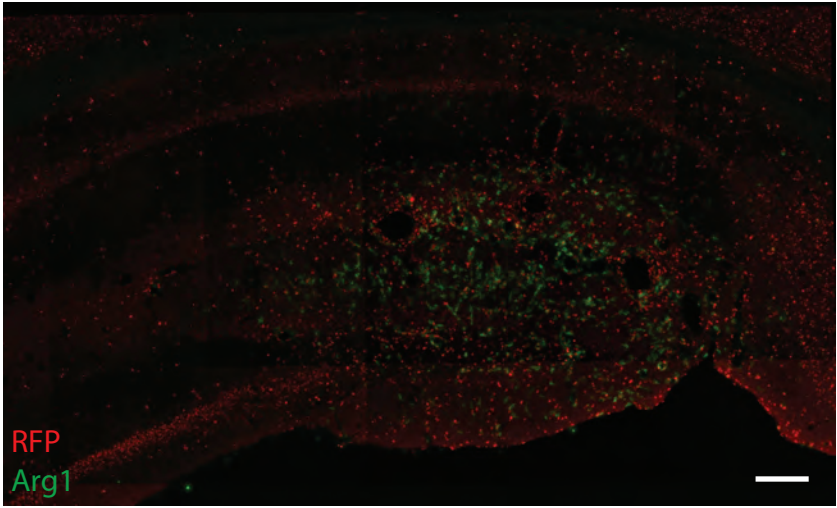

Supplement: Additional file 1: Figure S1. — Bone marrow chimeric mice demonstrate minimal overlap between peripheral donor RFP+ cells and Arg1+ cells. Representative image depicting RFP+ donor cells (red) and Arg1+ cells (green) 1 month after AAV-IL1β injection. Scale bar represents 50 μm. [file 12974_2015_411_MOESM1_ESM.pdf]
